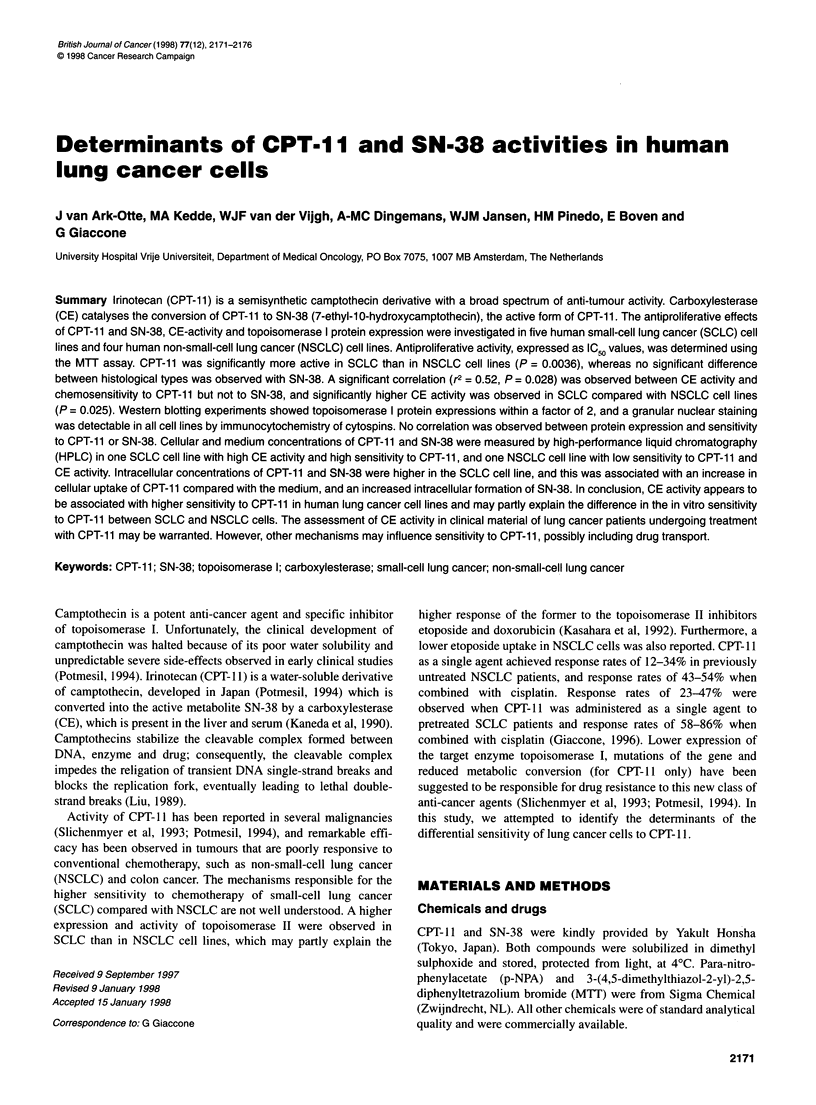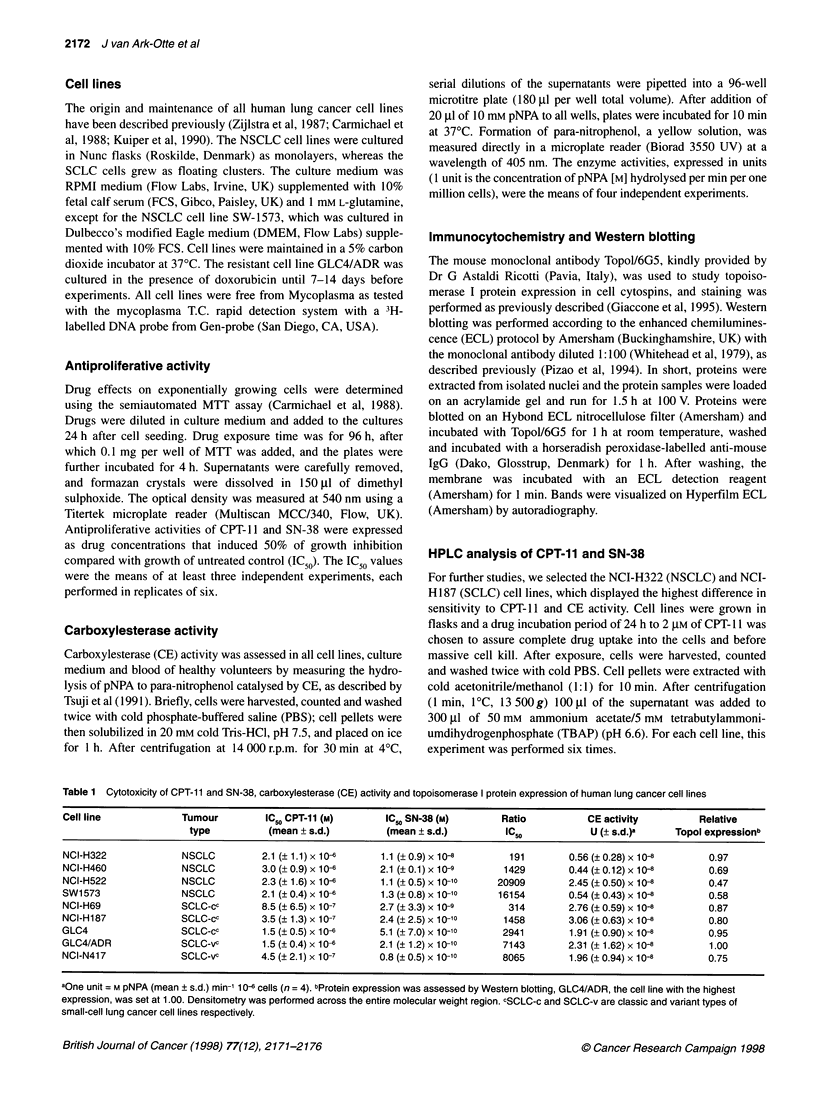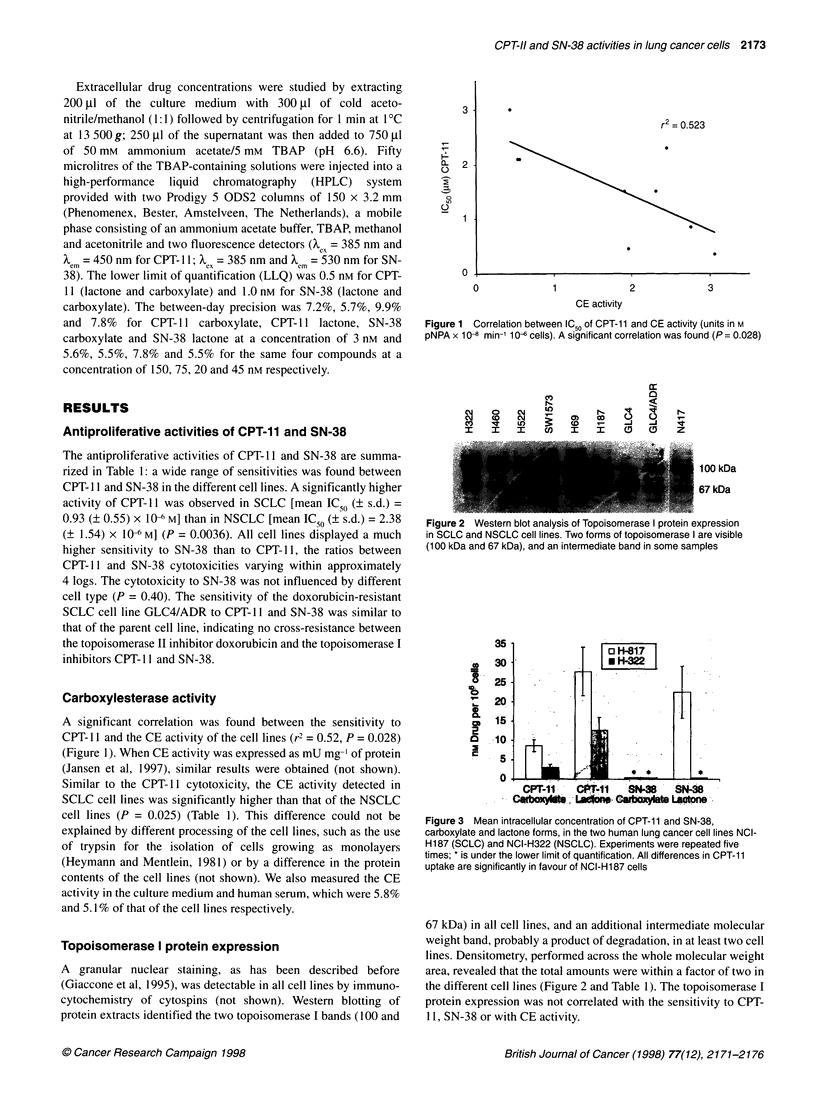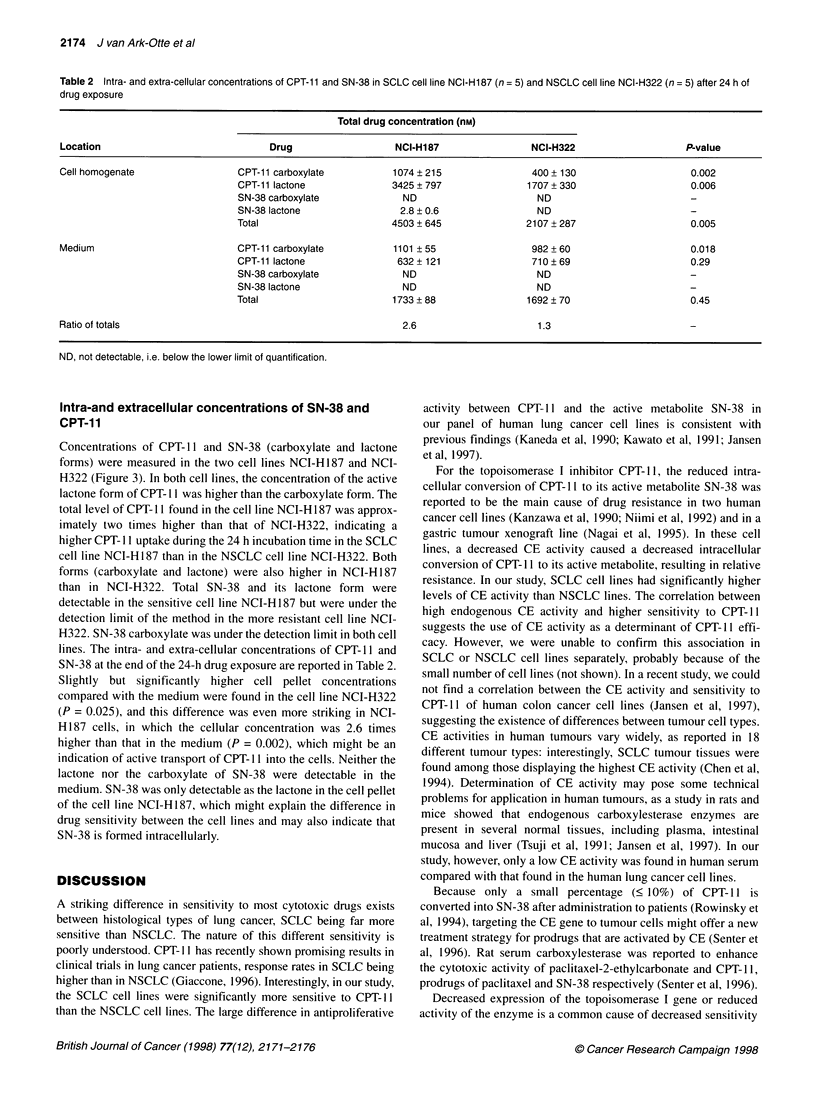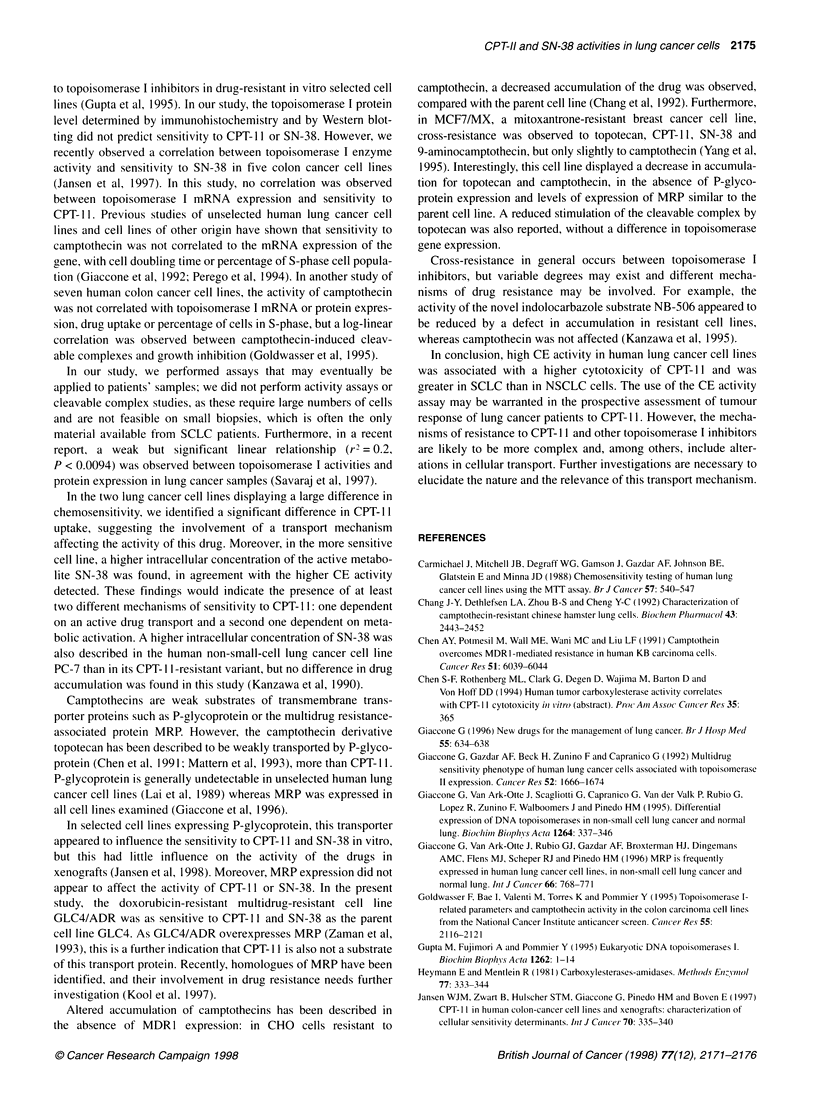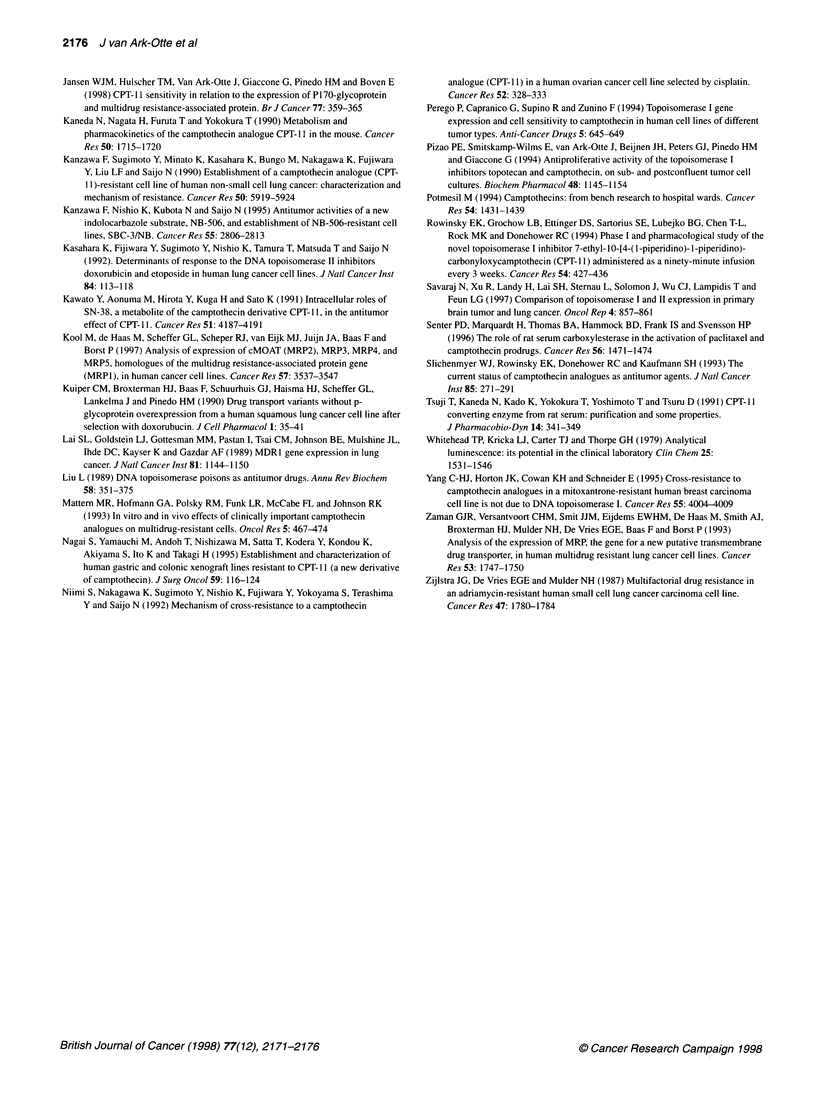# Determinants of CPT-11 and SN-38 activities in human lung cancer cells.

**DOI:** 10.1038/bjc.1998.362

**Published:** 1998-06

**Authors:** J. van Ark-Otte, M. A. Kedde, W. J. van der Vijgh, A. M. Dingemans, W. J. Jansen, H. M. Pinedo, E. Boven, G. Giaccone

**Affiliations:** University Hospital Vrije Universiteit, Department of Medical Oncology, Amsterdam, The Netherlands.

## Abstract

**Images:**


British Journal of Cancer (1998) 77(12), 2171–2176
© 1998 Cancer Research Campaign

### Determinants of CPT-11 and SN-38 activities in human lung cancer cells

**J van Ark-Otte, MA Kedde, WJF van der Vijgh, A-MC Dingemans, WJM Jansen, HM Pinedo, E Boven and G Giaccone**

University Hospital Vrije Universiteit, Department of Medical Oncology, PO Box 7075, 1007 MB Amsterdam, The Netherlands

**Summary** Irinotecan (CPT-11) is a semisynthetic camptothecin derivative with a broad spectrum of anti-tumour activity. Carboxylesterase (CE) catalyses the conversion of CPT-11 to SN-38 (7-ethyl-10-hydroxycamptothecin), the active form of CPT-11. The antiproliferative effects of CPT-11 and SN-38, CE-activity and topoisomerase I protein expression were investigated in five human small-cell lung cancer (SCLC) cell lines and four human non-small-cell lung cancer (NSCLC) cell lines. Antiproliferative activity, expressed as $IC_{50}$ values, was determined using the MTT assay. CPT-11 was significantly more active in SCLC than in NSCLC cell lines ($P = 0.0036$), whereas no significant difference between histological types was observed with SN-38. A significant correlation ($r^2 = 0.52$, $P = 0.028$) was observed between CE activity and chemosensitivity to CPT-11 but not to SN-38, and significantly higher CE activity was observed in SCLC compared with NSCLC cell lines ($P = 0.025$). Western blotting experiments showed topoisomerase I protein expressions within a factor of 2, and a granular nuclear staining was detectable in all cell lines by immunocytochemistry of cytospins. No correlation was observed between protein expression and sensitivity to CPT-11 or SN-38. Cellular and medium concentrations of CPT-11 and SN-38 were measured by high-performance liquid chromatography (HPLC) in one SCLC cell line with high CE activity and high sensitivity to CPT-11, and one NSCLC cell line with low sensitivity to CPT-11 and CE activity. Intracellular concentrations of CPT-11 and SN-38 were higher in the SCLC cell line, and this was associated with an increase in cellular uptake of CPT-11 compared with the medium, and an increased intracellular formation of SN-38. In conclusion, CE activity appears to be associated with higher sensitivity to CPT-11 in human lung cancer cell lines and may partly explain the difference in the in vitro sensitivity to CPT-11 between SCLC and NSCLC cells. The assessment of CE activity in clinical material of lung cancer patients undergoing treatment with CPT-11 may be warranted. However, other mechanisms may influence sensitivity to CPT-11, possibly including drug transport.

**Keywords:** CPT-11; SN-38; topoisomerase I; carboxylesterase; small-cell lung cancer; non-small-cell lung cancer

Camptothecin is a potent anti-cancer agent and specific inhibitor of topoisomerase I. Unfortunately, the clinical development of camptothecin was halted because of its poor water solubility and unpredictable severe side-effects observed in early clinical studies (Potmesil, 1994). Irinotecan (CPT-11) is a water-soluble derivative of camptothecin, developed in Japan (Potmesil, 1994) which is converted into the active metabolite SN-38 by a carboxylesterase (CE), which is present in the liver and serum (Kaneda et al, 1990). Camptothecins stabilize the cleavable complex formed between DNA, enzyme and drug; consequently, the cleavable complex impedes the religation of transient DNA single-strand breaks and blocks the replication fork, eventually leading to lethal double-strand breaks (Liu, 1989).

Activity of CPT-11 has been reported in several malignancies (Slichenmyer et al, 1993; Potmesil, 1994), and remarkable efficacy has been observed in tumours that are poorly responsive to conventional chemotherapy, such as non-small-cell lung cancer (NSCLC) and colon cancer. The mechanisms responsible for the higher sensitivity to chemotherapy of small-cell lung cancer (SCLC) compared with NSCLC are not well understood. A higher expression and activity of topoisomerase II were observed in SCLC than in NSCLC cell lines, which may partly explain the higher response of the former to the topoisomerase II inhibitors etoposide and doxorubicin (Kasahara et al, 1992). Furthermore, a lower etoposide uptake in NSCLC cells was also reported. CPT-11 as a single agent achieved response rates of 12–34% in previously untreated NSCLC patients, and response rates of 43–54% when combined with cisplatin. Response rates of 23–47% were observed when CPT-11 was administered as a single agent to pretreated SCLC patients and response rates of 58–86% when combined with cisplatin (Giaccone, 1996). Lower expression of the target enzyme topoisomerase I, mutations of the gene and reduced metabolic conversion (for CPT-11 only) have been suggested to be responsible for drug resistance to this new class of anti-cancer agents (Slichenmyer et al, 1993; Potmesil, 1994). In this study, we attempted to identify the determinants of the differential sensitivity of lung cancer cells to CPT-11.

*Received 9 September 1997*
*Revised 9 January 1998*
*Accepted 15 January 1998*

*Correspondence to:* G Giaccone

### MATERIALS AND METHODS

### Chemicals and drugs

CPT-11 and SN-38 were kindly provided by Yakult Honsha (Tokyo, Japan). Both compounds were solubilized in dimethyl sulphoxide and stored, protected from light, at 4°C. Para-nitrophenylacetate (p-NPA) and 3-(4,5-dimethylthiazol-2-yl)-2,5-diphenyltetrazolium bromide (MTT) were from Sigma Chemical (Zwijndrecht, NL). All other chemicals were of standard analytical quality and were commercially available.

### Cell lines

The origin and maintenance of all human lung cancer cell lines have been described previously (Zijlstra et al, 1987; Carmichael et al, 1988; Kuiper et al, 1990). The NSCLC cell lines were cultured in Nunc flasks (Roskilde, Denmark) as monolayers, whereas the SCLC cells grew as floating clusters. The culture medium was RPMI medium (Flow Labs, Irvine, UK) supplemented with 10% fetal calf serum (FCS, Gibco, Paisley, UK) and 1 mM L-glutamine, except for the NSCLC cell line SW-1573, which was cultured in Dulbecco's modified Eagle medium (DMEM, Flow Labs) supplemented with 10% FCS. Cell lines were maintained in a 5% carbon dioxide incubator at 37°C. The resistant cell line GLC4/ADR was cultured in the presence of doxorubicin until 7–14 days before experiments. All cell lines were free from Mycoplasma as tested with the mycoplasma T.C. rapid detection system with a $^{3}$H-labelled DNA probe from Gen-probe (San Diego, CA, USA).

### Antiproliferative activity

Drug effects on exponentially growing cells were determined using the semiautomated MTT assay (Carmichael et al, 1988). Drugs were diluted in culture medium and added to the cultures 24 h after cell seeding. Drug exposure time was for 96 h, after which 0.1 mg per well of MTT was added, and the plates were further incubated for 4 h. Supernatants were carefully removed, and formazan crystals were dissolved in 150 µl of dimethyl sulphoxide. The optical density was measured at 540 nm using a Titertek microplate reader (Multiscan MCC/340, Flow, UK). Antiproliferative activities of CPT-11 and SN-38 were expressed as drug concentrations that induced 50% of growth inhibition compared with growth of untreated control ($IC_{50}$). The $IC_{50}$ values were the means of at least three independent experiments, each performed in replicates of six.

### Carboxylesterase activity

Carboxylesterase (CE) activity was assessed in all cell lines, culture medium and blood of healthy volunteers by measuring the hydrolysis of pNPA to para-nitrophenol catalysed by CE, as described by Tsuji et al (1991). Briefly, cells were harvested, counted and washed twice with cold phosphate-buffered saline (PBS); cell pellets were then solubilized in 20 mM cold Tris-HCl, pH 7.5, and placed on ice for 1 h. After centrifugation at 14 000 r.p.m. for 30 min at 4°C, serial dilutions of the supernatants were pipetted into a 96-well microtitre plate (180 µl per well total volume). After addition of 20 µl of 10 mM pNPA to all wells, plates were incubated for 10 min at 37°C. Formation of para-nitrophenol, a yellow solution, was measured directly in a microplate reader (Biorad 3550 UV) at a wavelength of 405 nm. The enzyme activities, expressed in units (1 unit is the concentration of pNPA [M] hydrolysed per min per one million cells), were the means of four independent experiments.

### Immunocytochemistry and Western blotting

The mouse monoclonal antibody Topol/6G5, kindly provided by Dr G Astaldi Ricotti (Pavia, Italy), was used to study topoisomerase I protein expression in cell cytospins, and staining was performed as previously described (Giaccone et al, 1995). Western blotting was performed according to the enhanced chemiluminescence (ECL) protocol by Amersham (Buckinghamshire, UK) with the monoclonal antibody diluted 1:100 (Whitehead et al, 1979), as described previously (Pizao et al, 1994). In short, proteins were extracted from isolated nuclei and the protein samples were loaded on an acrylamide gel and run for 1.5 h at 100 V. Proteins were blotted on an Hybond ECL nitrocellulose filter (Amersham) and incubated with Topol/6G5 for 1 h at room temperature, washed and incubated with a horseradish peroxidase-labelled anti-mouse IgG (Dako, Glosstrup, Denmark) for 1 h. After washing, the membrane was incubated with an ECL detection reagent (Amersham) for 1 min. Bands were visualized on Hyperfilm ECL (Amersham) by autoradiography.

### HPLC analysis of CPT-11 and SN-38

For further studies, we selected the NCI-H322 (NSCLC) and NCI-H187 (SCLC) cell lines, which displayed the highest difference in sensitivity to CPT-11 and CE activity. Cell lines were grown in flasks and a drug incubation period of 24 h to 2 µM of CPT-11 was chosen to assure complete drug uptake into the cells and before massive cell kill. After exposure, cells were harvested, counted and washed twice with cold PBS. Cell pellets were extracted with cold acetonitrile/methanol (1:1) for 10 min. After centrifugation (1 min, 1°C, 13 500 *g*) 100 µl of the supernatant was added to 300 µl of 50 mM ammonium acetate/5 mM tetrabutylammoniumdihydrogenphosphate (TBAP) (pH 6.6). For each cell line, this experiment was performed six times.

**Table 1** Cytotoxicity of CPT-11 and SN-38, carboxylesterase (CE) activity and topoisomerase I protein expression of human lung cancer cell lines

| Cell line | Tumour type | $IC_{50}$ CPT-11 (M) (mean ± s.d.) | $IC_{50}$ SN-38 (M) (mean ± s.d.) | Ratio $IC_{50}$ | CE activity U (± s.d.)[a] | Relative Topol expression[b] |
|---|---|---|---|---|---|---|
| NCI-H322 | NSCLC | 2.1 (± 1.1) × $10^{-6}$ | 1.1 (± 0.9) × $10^{-8}$ | 191 | 0.56 (± 0.28) × $10^{-8}$ | 0.97 |
| NCI-H460 | NSCLC | 3.0 (± 0.9) × $10^{-6}$ | 2.1 (± 0.1) × $10^{-9}$ | 1429 | 0.44 (± 0.12) × $10^{-8}$ | 0.69 |
| NCI-H522 | NSCLC | 2.3 (± 1.6) × $10^{-6}$ | 1.1 (± 0.5) × $10^{-10}$ | 20909 | 2.45 (± 0.50) × $10^{-8}$ | 0.47 |
| SW1573 | NSCLC | 2.1 (± 0.4) × $10^{-6}$ | 1.3 (± 0.8) × $10^{-10}$ | 16154 | 0.54 (± 0.43) × $10^{-8}$ | 0.58 |
| NCI-H69 | SCLC-c[c] | 8.5 (± 6.5) × $10^{-7}$ | 2.7 (± 3.3) × $10^{-9}$ | 314 | 2.76 (± 0.59) × $10^{-8}$ | 0.87 |
| NCI-H187 | SCLC-c[c] | 3.5 (± 1.3) × $10^{-7}$ | 2.4 (± 2.5) × $10^{-10}$ | 1458 | 3.06 (± 0.63) × $10^{-8}$ | 0.80 |
| GLC4 | SCLC-c[c] | 1.5 (± 0.5) × $10^{-6}$ | 5.1 (± 7.0) × $10^{-10}$ | 2941 | 1.91 (± 0.90) × $10^{-8}$ | 0.95 |
| GLC4/ADR | SCLC-v[c] | 1.5 (± 0.4) × $10^{-6}$ | 2.1 (± 1.2) × $10^{-10}$ | 7143 | 2.31 (± 1.62) × $10^{-8}$ | 1.00 |
| NCI-N417 | SCLC-v[c] | 4.5 (± 2.1) × $10^{-7}$ | 0.8 (± 0.5) × $10^{-10}$ | 8065 | 1.96 (± 0.94) × $10^{-8}$ | 0.75 |

[a]One unit = M pNPA (mean ± s.d.) $min^{-1}$ $10^{-6}$ cells ($n$ = 4). [b]Protein expression was assessed by Western blotting, GLC4/ADR, the cell line with the highest expression, was set at 1.00. Densitometry was performed across the entire molecular weight region. [c]SCLC-c and SCLC-v are classic and variant types of small-cell lung cancer cell lines respectively.

© Cancer Research Campaign 1998

Extracellular drug concentrations were studied by extracting 200 µl of the culture medium with 300 µl of cold acetonitrile/methanol (1:1) followed by centrifugation for 1 min at 1°C at 13 500 *g*; 250 µl of the supernatant was then added to 750 µl of 50 mM ammonium acetate/5 mM TBAP (pH 6.6). Fifty microlitres of the TBAP-containing solutions were injected into a high-performance liquid chromatography (HPLC) system provided with two Prodigy 5 ODS2 columns of 150 × 3.2 mm (Phenomenex, Bester, Amstelveen, The Netherlands), a mobile phase consisting of an ammonium acetate buffer, TBAP, methanol and acetonitrile and two fluorescence detectors ($\lambda_{ex}$ = 385 nm and $\lambda_{em}$ = 450 nm for CPT-11; $\lambda_{ex}$ = 385 nm and $\lambda_{em}$ = 530 nm for SN-38). The lower limit of quantification (LLQ) was 0.5 nM for CPT-11 (lactone and carboxylate) and 1.0 nM for SN-38 (lactone and carboxylate). The between-day precision was 7.2%, 5.7%, 9.9% and 7.8% for CPT-11 carboxylate, CPT-11 lactone, SN-38 carboxylate and SN-38 lactone at a concentration of 3 nM and 5.6%, 5.5%, 7.8% and 5.5% for the same four compounds at a concentration of 150, 75, 20 and 45 nM respectively.

### RESULTS

### Antiproliferative activities of CPT-11 and SN-38

The antiproliferative activities of CPT-11 and SN-38 are summarized in Table 1: a wide range of sensitivities was found between CPT-11 and SN-38 in the different cell lines. A significantly higher activity of CPT-11 was observed in SCLC [mean $IC_{50}$ (± s.d.) = 0.93 (± 0.55) × $10^{-6}$ M] than in NSCLC [mean $IC_{50}$ (± s.d.) = 2.38 (± 1.54) × $10^{-6}$ M] ($P$ = 0.0036). All cell lines displayed a much higher sensitivity to SN-38 than to CPT-11, the ratios between CPT-11 and SN-38 cytotoxicities varying within approximately 4 logs. The cytotoxicity to SN-38 was not influenced by different cell type ($P$ = 0.40). The sensitivity of the doxorubicin-resistant SCLC cell line GLC4/ADR to CPT-11 and SN-38 was similar to that of the parent cell line, indicating no cross-resistance between the topoisomerase II inhibitor doxorubicin and the topoisomerase I inhibitors CPT-11 and SN-38.

### Carboxylesterase activity

A significant correlation was found between the sensitivity to CPT-11 and the CE activity of the cell lines ($r^2$ = 0.52, $P$ = 0.028) (Figure 1). When CE activity was expressed as mU $mg^{-1}$ of protein (Jansen et al, 1997), similar results were obtained (not shown). Similar to the CPT-11 cytotoxicity, the CE activity detected in SCLC cell lines was significantly higher than that of the NSCLC cell lines ($P$ = 0.025) (Table 1). This difference could not be explained by different processing of the cell lines, such as the use of trypsin for the isolation of cells growing as monolayers (Heymann and Mentlein, 1981) or by a difference in the protein contents of the cell lines (not shown). We also measured the CE activity in the culture medium and human serum, which were 5.8% and 5.1% of that of the cell lines respectively.

### Topoisomerase I protein expression

A granular nuclear staining, as has been described before (Giaccone et al, 1995), was detectable in all cell lines by immunocytochemistry of cytospins (not shown). Western blotting of protein extracts identified the two topoisomerase I bands (100 and 67 kDa) in all cell lines, and an additional intermediate molecular weight band, probably a product of degradation, in at least two cell lines. Densitometry, performed across the whole molecular weight area, revealed that the total amounts were within a factor of two in the different cell lines (Figure 2 and Table 1). The topoisomerase I protein expression was not correlated with the sensitivity to CPT-11, SN-38 or with CE activity.

**Figure 1** Correlation between $IC_{50}$ of CPT-11 and CE activity (units in M pNPA × $10^{-8}$ $min^{-1}$ $10^{-6}$ cells). A significant correlation was found ($P$ = 0.028)

**Figure 2** Western blot analysis of Topoisomerase I protein expression in SCLC and NSCLC cell lines. Two forms of topoisomerase I are visible (100 kDa and 67 kDa), and an intermediate band in some samples

**Figure 3** Mean intracellular concentration of CPT-11 and SN-38, carboxylate and lactone forms, in the two human lung cancer cell lines NCI-H187 (SCLC) and NCI-H322 (NSCLC). Experiments were repeated five times; * is under the lower limit of quantification. All differences in CPT-11 uptake are significantly in favour of NCI-H187 cells

**Table 2** Intra- and extra-cellular concentrations of CPT-11 and SN-38 in SCLC cell line NCI-H187 ($n$ = 5) and NSCLC cell line NCI-H322 ($n$ = 5) after 24 h of drug exposure

| | | Total drug concentration (nM) | | |
|---|---|---|---|---|
| Location | Drug | NCI-H187 | NCI-H322 | P-value |
| Cell homogenate | CPT-11 carboxylate | 1074 ± 215 | 400 ± 130 | 0.002 |
| | CPT-11 lactone | 3425 ± 797 | 1707 ± 330 | 0.006 |
| | SN-38 carboxylate | ND | ND | – |
| | SN-38 lactone | 2.8 ± 0.6 | ND | – |
| | Total | 4503 ± 645 | 2107 ± 287 | 0.005 |
| Medium | CPT-11 carboxylate | 1101 ± 55 | 982 ± 60 | 0.018 |
| | CPT-11 lactone | 632 ± 121 | 710 ± 69 | 0.29 |
| | SN-38 carboxylate | ND | ND | – |
| | SN-38 lactone | ND | ND | – |
| | Total | 1733 ± 88 | 1692 ± 70 | 0.45 |
| Ratio of totals | | 2.6 | 1.3 | – |

ND, not detectable, i.e. below the lower limit of quantification.

### Intra-and extracellular concentrations of SN-38 and CPT-11

Concentrations of CPT-11 and SN-38 (carboxylate and lactone forms) were measured in the two cell lines NCI-H187 and NCI-H322 (Figure 3). In both cell lines, the concentration of the active lactone form of CPT-11 was higher than the carboxylate form. The total level of CPT-11 found in the cell line NCI-H187 was approximately two times higher than that of NCI-H322, indicating a higher CPT-11 uptake during the 24 h incubation time in the SCLC cell line NCI-H187 than in the NSCLC cell line NCI-H322. Both forms (carboxylate and lactone) were also higher in NCI-H187 than in NCI-H322. Total SN-38 and its lactone form were detectable in the sensitive cell line NCI-H187 but were under the detection limit of the method in the more resistant cell line NCI-H322. SN-38 carboxylate was under the detection limit in both cell lines. The intra- and extra-cellular concentrations of CPT-11 and SN-38 at the end of the 24-h drug exposure are reported in Table 2. Slightly but significantly higher cell pellet concentrations compared with the medium were found in the cell line NCI-H322 ($P$ = 0.025), and this difference was even more striking in NCI-H187 cells, in which the cellular concentration was 2.6 times higher than that in the medium ($P$ = 0.002), which might be an indication of active transport of CPT-11 into the cells. Neither the lactone nor the carboxylate of SN-38 were detectable in the medium. SN-38 was only detectable as the lactone in the cell pellet of the cell line NCI-H187, which might explain the difference in drug sensitivity between the cell lines and may also indicate that SN-38 is formed intracellularly.

### DISCUSSION

A striking difference in sensitivity to most cytotoxic drugs exists between histological types of lung cancer, SCLC being far more sensitive than NSCLC. The nature of this different sensitivity is poorly understood. CPT-11 has recently shown promising results in clinical trials in lung cancer patients, response rates in SCLC being higher than in NSCLC (Giaccone, 1996). Interestingly, in our study, the SCLC cell lines were significantly more sensitive to CPT-11 than the NSCLC cell lines. The large difference in antiproliferative activity between CPT-11 and the active metabolite SN-38 in our panel of human lung cancer cell lines is consistent with previous findings (Kaneda et al, 1990; Kawato et al, 1991; Jansen et al, 1997).

For the topoisomerase I inhibitor CPT-11, the reduced intracellular conversion of CPT-11 to its active metabolite SN-38 was reported to be the main cause of drug resistance in two human cancer cell lines (Kanzawa et al, 1990; Niimi et al, 1992) and in a gastric tumour xenograft line (Nagai et al, 1995). In these cell lines, a decreased CE activity caused a decreased intracellular conversion of CPT-11 to its active metabolite, resulting in relative resistance. In our study, SCLC cell lines had significantly higher levels of CE activity than NSCLC lines. The correlation between high endogenous CE activity and higher sensitivity to CPT-11 suggests the use of CE activity as a determinant of CPT-11 efficacy. However, we were unable to confirm this association in SCLC or NSCLC cell lines separately, probably because of the small number of cell lines (not shown). In a recent study, we could not find a correlation between the CE activity and sensitivity to CPT-11 of human colon cancer cell lines (Jansen et al, 1997), suggesting the existence of differences between tumour cell types. CE activities in human tumours vary widely, as reported in 18 different tumour types: interestingly, SCLC tumour tissues were found among those displaying the highest CE activity (Chen et al, 1994). Determination of CE activity may pose some technical problems for application in human tumours, as a study in rats and mice showed that endogenous carboxylesterase enzymes are present in several normal tissues, including plasma, intestinal mucosa and liver (Tsuji et al, 1991; Jansen et al, 1997). In our study, however, only a low CE activity was found in human serum compared with that found in the human lung cancer cell lines.

Because only a small percentage (≤ 10%) of CPT-11 is converted into SN-38 after administration to patients (Rowinsky et al, 1994), targeting the CE gene to tumour cells might offer a new treatment strategy for prodrugs that are activated by CE (Senter et al, 1996). Rat serum carboxylesterase was reported to enhance the cytotoxic activity of paclitaxel-2-ethylcarbonate and CPT-11, prodrugs of paclitaxel and SN-38 respectively (Senter et al, 1996).

Decreased expression of the topoisomerase I gene or reduced activity of the enzyme is a common cause of decreased sensitivity

© Cancer Research Campaign 1998

to topoisomerase I inhibitors in drug-resistant in vitro selected cell lines (Gupta et al, 1995). In our study, the topoisomerase I protein level determined by immunohistochemistry and by Western blotting did not predict sensitivity to CPT-11 or SN-38. However, we recently observed a correlation between topoisomerase I enzyme activity and sensitivity to SN-38 in five colon cancer cell lines (Jansen et al, 1997). In this study, no correlation was observed between topoisomerase I mRNA expression and sensitivity to CPT-11. Previous studies of unselected human lung cancer cell lines and cell lines of other origin have shown that sensitivity to camptothecin was not correlated to the mRNA expression of the gene, with cell doubling time or percentage of S-phase cell population (Giaccone et al, 1992; Perego et al, 1994). In another study of seven human colon cancer cell lines, the activity of camptothecin was not correlated with topoisomerase I mRNA or protein expression, drug uptake or percentage of cells in S-phase, but a log-linear correlation was observed between camptothecin-induced cleavable complexes and growth inhibition (Goldwasser et al, 1995).

In our study, we performed assays that may eventually be applied to patients' samples; we did not perform activity assays or cleavable complex studies, as these require large numbers of cells and are not feasible on small biopsies, which is often the only material available from SCLC patients. Furthermore, in a recent report, a weak but significant linear relationship ($r^2 = 0.2$, $P < 0.0094$) was observed between topoisomerase I activities and protein expression in lung cancer samples (Savaraj et al, 1997).

In the two lung cancer cell lines displaying a large difference in chemosensitivity, we identified a significant difference in CPT-11 uptake, suggesting the involvement of a transport mechanism affecting the activity of this drug. Moreover, in the more sensitive cell line, a higher intracellular concentration of the active metabolite SN-38 was found, in agreement with the higher CE activity detected. These findings would indicate the presence of at least two different mechanisms of sensitivity to CPT-11: one dependent on an active drug transport and a second one dependent on metabolic activation. A higher intracellular concentration of SN-38 was also described in the human non-small-cell lung cancer cell line PC-7 than in its CPT-11-resistant variant, but no difference in drug accumulation was found in this study (Kanzawa et al, 1990).

Camptothecins are weak substrates of transmembrane transporter proteins such as P-glycoprotein or the multidrug resistance-associated protein MRP. However, the camptothecin derivative topotecan has been described to be weakly transported by P-glycoprotein (Chen et al, 1991; Mattern et al, 1993), more than CPT-11. P-glycoprotein is generally undetectable in unselected human lung cancer cell lines (Lai et al, 1989) whereas MRP was expressed in all cell lines examined (Giaccone et al, 1996).

In selected cell lines expressing P-glycoprotein, this transporter appeared to influence the sensitivity to CPT-11 and SN-38 in vitro, but this had little influence on the activity of the drugs in xenografts (Jansen et al, 1998). Moreover, MRP expression did not appear to affect the activity of CPT-11 or SN-38. In the present study, the doxorubicin-resistant multidrug-resistant cell line GLC4/ADR was as sensitive to CPT-11 and SN-38 as the parent cell line GLC4. As GLC4/ADR overexpresses MRP (Zaman et al, 1993), this is a further indication that CPT-11 is also not a substrate of this transport protein. Recently, homologues of MRP have been identified, and their involvement in drug resistance needs further investigation (Kool et al, 1997).

Altered accumulation of camptothecins has been described in the absence of MDR1 expression: in CHO cells resistant to camptothecin, a decreased accumulation of the drug was observed, compared with the parent cell line (Chang et al, 1992). Furthermore, in MCF7/MX, a mitoxantrone-resistant breast cancer cell line, cross-resistance was observed to topotecan, CPT-11, SN-38 and 9-aminocamptothecin, but only slightly to camptothecin (Yang et al, 1995). Interestingly, this cell line displayed a decrease in accumulation for topotecan and camptothecin, in the absence of P-glycoprotein expression and levels of expression of MRP similar to the parent cell line. A reduced stimulation of the cleavable complex by topotecan was also reported, without a difference in topoisomerase gene expression.

Cross-resistance in general occurs between topoisomerase I inhibitors, but variable degrees may exist and different mechanisms of drug resistance may be involved. For example, the activity of the novel indolocarbazole substrate NB-506 appeared to be reduced by a defect in accumulation in resistant cell lines, whereas camptothecin was not affected (Kanzawa et al, 1995).

In conclusion, high CE activity in human lung cancer cell lines was associated with a higher cytotoxicity of CPT-11 and was greater in SCLC than in NSCLC cells. The use of the CE activity assay may be warranted in the prospective assessment of tumour response of lung cancer patients to CPT-11. However, the mechanisms of resistance to CPT-11 and other topoisomerase I inhibitors are likely to be more complex and, among others, include alterations in cellular transport. Further investigations are necessary to elucidate the nature and the relevance of this transport mechanism.

### REFERENCES

Carmichael J, Mitchell JB, Degraff WG, Gamson J, Gazdar AF, Johnson BE, Glatstein E and Minna JD (1988) Chemosensitivity testing of human lung cancer cell lines using the MTT assay. *Br J Cancer* **57**: 540–547

Chang J-Y, Dethlefsen LA, Zhou B-S and Cheng Y-C (1992) Characterization of camptothecin-resistant chinese hamster lung cells. *Biochem Pharmacol* **43**: 2443–2452

Chen AY, Potmesil M, Wall ME, Wani MC and Liu LF (1991) Camptothein overcomes MDR1-mediated resistance in human KB carcinoma cells. *Cancer Res* **51**: 6039–6044

Chen S-F, Rothenberg ML, Clark G, Degen D, Wajima M, Barton D and Von Hoff DD (1994) Human tumor carboxylesterase activity correlates with CPT-11 cytotoxicity *in vitro* (abstract). *Proc Am Assoc Cancer Res* **35**: 365

Giaccone G (1996) New drugs for the management of lung cancer. *Br J Hosp Med* **55**: 634–638

Giaccone G, Gazdar AF, Beck H, Zunino F and Capranico G (1992) Multidrug sensitivity phenotype of human lung cancer cells associated with topoisomerase II expression. *Cancer Res* **52**: 1666–1674

Giaccone G, Van Ark-Otte J, Scagliotti G, Capranico G, Van der Valk P, Rubio G, Lopez R, Zunino F, Walboomers J and Pinedo HM (1995). Differential expression of DNA topoisomerases in non-small cell lung cancer and normal lung. *Biochim Biophys Acta* **1264**: 337–346

Giaccone G, Van Ark-Otte J, Rubio GJ, Gazdar AF, Broxterman HJ, Dingemans AMC, Flens MJ, Scheper RJ and Pinedo HM (1996) MRP is frequently expressed in human lung cancer cell lines, in non-small cell lung cancer and normal lung. *Int J Cancer* **66**: 768–771

Goldwasser F, Bae I, Valenti M, Torres K and Pommier Y (1995) Topoisomerase I-related parameters and camptothecin activity in the colon carcinoma cell lines from the National Cancer Institute anticancer screen. *Cancer Res* **55**: 2116–2121

Gupta M, Fujimori A and Pommier Y (1995) Eukaryotic DNA topoisomerases I. *Biochim Biophys Acta* **1262**: 1–14

Heymann E and Mentlein R (1981) Carboxylesterases-amidases. *Methods Enzymol* **77**: 333–344

Jansen WJM, Zwart B, Hulscher STM, Giaccone G, Pinedo HM and Boven E (1997) CPT-11 in human colon-cancer cell lines and xenografts: characterization of cellular sensitivity determinants. *Int J Cancer* **70**: 335–340

© Cancer Research Campaign 1998 

Jansen WJM, Hulscher TM, Van Ark-Otte J, Giaccone G, Pinedo HM and Boven E (1998) CPT-11 sensitivity in relation to the expression of P170-glycoprotein and multidrug resistance-associated protein. *Br J Cancer* **77**: 359–365

Kaneda N, Nagata H, Furuta T and Yokokura T (1990) Metabolism and pharmacokinetics of the camptothecin analogue CPT-11 in the mouse. *Cancer Res* **50**: 1715–1720

Kanzawa F, Sugimoto Y, Minato K, Kasahara K, Bungo M, Nakagawa K, Fujiwara Y, Liu LF and Saijo N (1990) Establishment of a camptothecin analogue (CPT-11)-resistant cell line of human non-small cell lung cancer: characterization and mechanism of resistance. *Cancer Res* **50**: 5919–5924

Kanzawa F, Nishio K, Kubota N and Saijo N (1995) Antitumor activities of a new indolocarbazole substrate, NB-506, and establishment of NB-506-resistant cell lines, SBC-3/NB. *Cancer Res* **55**: 2806–2813

Kasahara K, Fijiwara Y, Sugimoto Y, Nishio K, Tamura T, Matsuda T and Saijo N (1992). Determinants of response to the DNA topoisomerase II inhibitors doxorubicin and etoposide in human lung cancer cell lines. *J Natl Cancer Inst* **84**: 113–118

Kawato Y, Aonuma M, Hirota Y, Kuga H and Sato K (1991) Intracellular roles of SN-38, a metabolite of the camptothecin derivative CPT-11, in the antitumor effect of CPT-11. *Cancer Res* **51**: 4187–4191

Kool M, de Haas M, Scheffer GL, Scheper RJ, van Eijk MJ, Juijn JA, Baas F and Borst P (1997) Analysis of expression of cMOAT (MRP2), MRP3, MRP4, and MRP5, homologues of the multidrug resistance-associated protein gene (MRP1), in human cancer cell lines. *Cancer Res* **57**: 3537–3547

Kuiper CM, Broxterman HJ, Baas F, Schuurhuis GJ, Haisma HJ, Scheffer GL, Lankelma J and Pinedo HM (1990) Drug transport variants without p-glycoprotein overexpression from a human squamous lung cancer cell line after selection with doxorubicin. *J Cell Pharmacol* **1**: 35–41

Lai SL, Goldstein LJ, Gottesman MM, Pastan I, Tsai CM, Johnson BE, Mulshine JL, Ihde DC, Kayser K and Gazdar AF (1989) MDR1 gene expression in lung cancer. *J Natl Cancer Inst* **81**: 1144–1150

Liu L (1989) DNA topoisomerase poisons as antitumor drugs. *Annu Rev Biochem* **58**: 351–375

Mattern MR, Hofmann GA, Polsky RM, Funk LR, McCabe FL and Johnson RK (1993) In vitro and in vivo effects of clinically important camptothecin analogues on multidrug-resistant cells. *Oncol Res* **5**: 467–474

Nagai S, Yamauchi M, Andoh T, Nishizawa M, Satta T, Kodera Y, Kondou K, Akiyama S, Ito K and Takagi H (1995) Establishment and characterization of human gastric and colonic xenograft lines resistant to CPT-11 (a new derivative of camptothecin). *J Surg Oncol* **59**: 116–124

Niimi S, Nakagawa K, Sugimoto Y, Nishio K, Fujiwara Y, Yokoyama S, Terashima Y and Saijo N (1992) Mechanism of cross-resistance to a camptothecin analogue (CPT-11) in a human ovarian cancer cell line selected by cisplatin. *Cancer Res* **52**: 328–333

Perego P, Capranico G, Supino R and Zunino F (1994) Topoisomerase I gene expression and cell sensitivity to camptothecin in human cell lines of different tumor types. *Anti-Cancer Drugs* **5**: 645–649

Pizao PE, Smitskamp-Wilms E, van Ark-Otte J, Beijnen JH, Peters GJ, Pinedo HM and Giaccone G (1994) Antiproliferative activity of the topoisomerase I inhibitors topotecan and camptothecin, on sub- and postconfluent tumor cell cultures. *Biochem Pharmacol* **48**: 1145–1154

Potmesil M (1994) Camptothecins: from bench research to hospital wards. *Cancer Res* **54**: 1431–1439

Rowinsky EK, Grochow LB, Ettinger DS, Sartorius SE, Lubejko BG, Chen T-L, Rock MK and Donehower RC (1994) Phase I and pharmacological study of the novel topoisomerase I inhibitor 7-ethyl-10-[4-(1-piperidino)-1-piperidino)-carbonyloxycamptothecin (CPT-11) administered as a ninety-minute infusion every 3 weeks. *Cancer Res* **54**: 427–436

Savaraj N, Xu R, Landy H, Lai SH, Sternau L, Solomon J, Wu CJ, Lampidis T and Feun LG (1997) Comparison of topoisomerase I and II expression in primary brain tumor and lung cancer. *Oncol Rep* **4**: 857–861

Senter PD, Marquardt H, Thomas BA, Hammock BD, Frank IS and Svensson HP (1996) The role of rat serum carboxylesterase in the activation of paclitaxel and camptothecin prodrugs. *Cancer Res* **56**: 1471–1474

Slichenmyer WJ, Rowinsky EK, Donehower RC and Kaufmann SH (1993) The current status of camptothecin analogues as antitumor agents. *J Natl Cancer Inst* **85**: 271–291

Tsuji T, Kaneda N, Kado K, Yokokura T, Yoshimoto T and Tsuru D (1991) CPT-11 converting enzyme from rat serum: purification and some properties. *J Pharmacobio-Dyn* **14**: 341–349

Whitehead TP, Kricka LJ, Carter TJ and Thorpe GH (1979) Analytical luminescence: its potential in the clinical laboratory *Clin Chem* **25**: 1531–1546

Yang C-HJ, Horton JK, Cowan KH and Schneider E (1995) Cross-resistance to camptothecin analogues in a mitoxantrone-resistant human breast carcinoma cell line is not due to DNA topoisomerase I. *Cancer Res* **55**: 4004–4009

Zaman GJR, Versantvoort CHM, Smit JJM, Eijdems EWHM, De Haas M, Smith AJ, Broxterman HJ, Mulder NH, De Vries EGE, Baas F and Borst P (1993) Analysis of the expression of MRP, the gene for a new putative transmembrane drug transporter, in human multidrug resistant lung cancer cell lines. *Cancer Res* **53**: 1747–1750

Zijlstra JG, De Vries EGE and Mulder NH (1987) Multifactorial drug resistance in an adriamycin-resistant human small cell lung cancer carcinoma cell line. *Cancer Res* **47**: 1780–1784

© Cancer Research Campaign 1998